# Pre-travel malaria chemoprophylaxis counselling in a public travel medicine clinic in São Paulo, Brazil

**DOI:** 10.1186/s12936-017-1713-3

**Published:** 2017-02-07

**Authors:** Tânia do Socorro Souza Chaves, Wuelton Marcelo Monteiro, Jessé Reis Alves, Marcus Lacerda, Marta Heloisa Lopes

**Affiliations:** 1 Parasitology Section, Evandro Chagas Institute, Rodovia BR-316, Km 7 s/n, Levilândia, Ananindeua, PA 67030-300 Brazil; 2 Travel Medicine Division, Emilio Ribas Institute of Infectious Diseases, Av. Doutor Arnaldo 165, Cerqueira César, São Paulo, 01246-900 Brazil; 3 Departamento de Doenças Infecciosas, Faculdade de Medicina da Universidade de São Paulo, Av. Dr. Arnaldo 455, Cerqueira César, São Paulo, SP 01246-903 Brazil; 4 Fundação de Medicina Tropical Dr. Heitor Vieira Dourado, Manaus, Brazil; 5 Universidade do Estado do Amazonas, Manaus, Brazil; 6 Instituto Leônidas & Maria Deane (Fiocruz-Amazônia), Manaus, Brazil; 7 Disciplina de Doenças Infecciosas e Parasitárias, Faculdade de Medicina, Universidade Federal do Pará, Av. Generalíssimo Deodoro, 01 - Umarizal, Belém, PA 66050-160 Brazil

**Keywords:** Malaria prevention, Malaria chemoprophylaxis, Travel medicine, *Plasmodium vivax*, *Plasmodium falciparum*

## Abstract

**Background:**

Malaria is one of the most prevalent parasitic diseases in the world and represents a threat to travellers visiting endemic areas. Chemoprophylaxis is the prevention measure used in travel medicine, avoiding clinical manifestations and protecting against the development of severe disease and death.

**Methods:**

Retrospective and descriptive analysis of malaria prevention data in travellers was recorded from a travel medicine clinic in São Paulo, Brazil, between January 2006 and December 2010. All the medical records of travellers, who had travelled to areas with risk of disease transmission, including Brazil, were analysed. Demographic characteristics of travellers, travel details and recommendations for preventing malaria were also seen.

**Results:**

During the study period, 2836 pre-travel consultations were carried out on 2744 individuals (92 were consulted twice). The most common reasons for travelling were tourism and work. The most common destinations were Africa (24.5%), Europe (21.2%), Asia (16.6%) and locations within Brazil (14.9%). In general prophylaxis against malaria was recommended in 10.3% of all the consultations. African destinations vs Asian, Brazilian and other destinations and length of stay ≤30 days were independently associated with the higher odds of chemoprophylaxis recommendation after the logistic regression.

**Conclusion:**

The prophylaxis against malaria was recommended in 10.3% of the consultations. The authors believe that a coherent measure of malaria prevention in Brazil and for international travellers would be to recommend for all parts of the North Brazil, avoidance of mosquito bites and immediate consultation of a physician in case of fever during or after the journey is recommended.

## Background

Malaria is a preventable, diagnosable and treatable disease. With innovation and roll out of interventions there are fewer people dying from malaria now that in any other historical period. During 2015, there were an estimated 214 million cases of malaria and an estimated 438,000 deaths (range 236,000–635,000) globally [1].

The burden of malaria among residents in Latin America has been declining in recent years. The number of confirmed malaria cases in the region decreased from 1.2 million in 2000 to 427,000 cases in 2013. Reductions of >75% in the incidence of microscopically-confirmed malaria cases were reported in 13 out of 21 countries. Brazil and Colombia are on track to achieve a 75% decrease in case incidence by 2015, which would predominantly concern *Plasmodium vivax* malaria transmission [1]. Brazilian Amazon concentrates 99.8% of malaria cases in the country, a scenario in which the *P. vivax* (84%) is the prevalent species. Currently, the states of Acre, Amazonas, Pará, Amapá, Roraima and Rondônia present the greatest risks of transmission of *Plasmodium falciparum*, according to National Malaria Control Programme (PNCM) data from the Brazilian Ministry of Health [2].

**Fig. 1:**
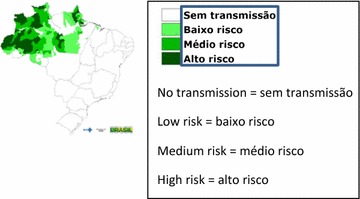
Areas at risk of malária transmission in Brazil

The malaria in the North of Brazil is dynamic and with heterogeneous occurrence, therefore, the risk of malaria transmission in the Brazilian Amazon is not homogeneous (Fig. 1). Urban malaria is limited to a few cities, especially in their suburbs which usually are not visited by tourists. Additionally, malaria in this region is often observed in gold mining areas and surrounding settlements, posing high risk to domestic travelers, which go to the areas looking for new job opportunities. For many years there has been no transmission of malaria in the most sought after tourist destinations such as Belém, Santarém, Alter do Chão, cities of the State of Pará.

Approximately 125 million international travellers per year will visit areas where there is a risk of malaria transmission, 10,000–30,000 of them will fall sick with malaria and between 1 and 4% of travellers who acquire falciparum malaria will die [3].

As there is no vaccine against malaria, prevention of disease consists of the person protective measures against mosquito bites and chemoprophylaxis. Chemoprophylaxis remains one of the main therapeutic intervention to prevent malaria-related severity and mortality [4, 5]. Malaria chemoprophylaxis has to be taken regularly to be effective and previous studies have shown poor compliance to be a common problem [6–9], with a considerable variation in efficacy comparing areas with different transmission risks [9]. Because travellers may be persuaded to stop taking medication by peer pressure, not seeing mosquitoes, and adverse reactions to medications, clinicians should be prepared to address these barriers [10]. Especially for long-term travellers, malaria prevention is a complex issue and requires expert and individualized advice from travel medicine specialists [11]. In the malaria elimination era, knowledge and practices regarding malaria chemoprophylaxis among health professional and travellers in malaria endemic areas is also important for preventing reintroduction of malaria to malaria-free zones [12].

Decisions on malaria chemoprophylaxis are complicated by a lack of standardized recommendations, controversies, and misconceptions [13]. Because of recent declining malaria transmission in Latin America, some researchers and authorities have not recommended chemoprophylaxis for most travellers to this region [14, 15]. In Brazil, malaria prevention among travellers is an issue that leads to discussions and controversies between malariologists and policy-makers. Mostly, chemoprophylaxis recommendation is reserved for specific situations in which the risk of becoming ill of severe *P. falciparum* malaria is greater than the risk of severe adverse events related to the use of chemoprophylatic drugs. Furthermore, there is a concern by the authorities that an amplified chemoprophylaxis could contribute to the emergence of resistance to anti-malarial treatments used in Brazil [15]. There are a range of suitable options for chemoprophylaxis in Latin America, including atovaquone-proguanil, doxycycline, mefloquine, and, in selected areas, chloroquine [16]. Currently, doxycycline and chloroquine are the drug regimens available for chemoprophylaxis in Brazil [15].

The aim of this study was to describe the pre-travel recommendation of malaria chemoprophylaxis in a free public clinic travel medicine in the city of São Paulo, Brazil. The results presented in this study do not represent the overall Brazilian travelling population.

## Methods

### Participants and procedures

This was a retrospective, descriptive study of the pre-travel counselling provided between January 2006 and December 2010 at the Travel Medicine Clinic of the *Instituto de Infectologia Emílio Ribas*, located in the city of São Paulo, Brazil [17]. All the medical records of individuals who sought pre-travel health advice during the study period were analysed. Data related to the socio-demographic characteristics (gender, age, schooling), clinical data (presence of any immunosuppressive condition, HIV infection, diabetes mellitus and depression), ways by which travellers became aware of the travel medicine services, travel characteristics (reason of the travel, destinations, accommodation type, travel length) and malaria chemoprophylaxis prescription (yes/no and prescribed drug) were retrieved.

### Statistical analysis

Only variables presenting completeness higher than 80% were analysed. Data were analysed using SPSS version 21.0 for Windows (SPSS Inc. Chicago, IL, USA). Proportions of individuals for which they were recommended chemoprophylaxis were compared by Chi square test (corrected by Fisher’ test if necessary); differences were considered statistically significant for p < 0.05. The crude Odds Ratio (OR) with its respective 95% Confidence Interval (95% CI) was determined considering chemoprophylaxis recommendation as the dependent variables. Logistic regression was used for the multivariable analyses and the adjusted ORs (AOR) with 95% CI were also calculated. All variables associated with the outcomes at a significance level of p < 0.20 in the univariable analysis were included in the multivariable analysis. Statistical significance was considered if p < 0.05 in the Hosmer–Lemeshow goodness-of-fit test.

### Ethical clearance

The study was approved by the Ethics in Medical Research Committee of the *Instituto de Infectologia Emílio Ribas* (09/12) and by the Research Ethics Committee of the *Hospital das Clínicas*, School of Medicine, University of São Paulo (311/12), also located in the city of São Paulo, Brazil.

## Results

### Population characteristics

Among the 2744 travellers who sought travel medicine consultations between January 2006 and December 2010, there were 2836 pre-travel consultations, since 92 of those travellers were attended more than once in the study period. Regarding gender, 51.1% of the attended population were female. Most of the travellers (54.2%) were aged between 18 and 34 years old, with a mean age of 34.7 years and a median of 31.3 years. A total of 75.6% had >11 years of schooling. In relation to clinical variables, any immunosuppressive condition was reported in 2.4%, HIV infection in 1.2%, diabetes mellitus in 1.3% and depression in 3.3% of the study population (Table 1). When travellers were asked how they had become aware of the travel medicine service, the most common responses were from the suggestion of friends, health professionals and electronic media.

**Table 1:** Socio-demographic and clinical characteristics of the population assisted at the Instituto Emílio Ribas travel clinics, São Paulo, Brazil, between January 2006 and December 2010

Variable (completeness)	N	%
*Gender (100%)*		
Male	1343	48.9
Female	1401	51.1
*Age (years; 100%)*		
0–9	66	2.4
10–17	90	3.3
18–24	522	19.0
25–34	966	35.2
35–44	486	17.7
45–59	457	16.7
≥60	157	5.7
Mean (standard deviation)	34.7 (±14.0)	–
Median (interquartile range)	31.3 (0.1–91.5)	–
*Schooling (years; 90.3%)*		
≥12	1872	75.6
8–11	472	19.0
<8	133	5.4
*Immunosuppressive condition (100%)*		
No	2678	97.6
Yes	66	2.4
*HIV infection (100%)*		
No	2711	98.8
Yes	33	1.2
*Diabetes mellitus (100%)*		
No	2708	98.7
Yes	36	1.3
*Depression (100%)*		
No	2653	96.7
Yes	91	3.3

### Travel characteristics

Among the reasons for travelling, study or work (52.7%) and tourism (36.1%) prevailed. The most common destinations were Africa (24.5%), Europe (21.2%), locations within Brazil (15.1%) and Asia (14.1%). Most of the individuals travelling within Brazil had as destinations the North (51.4%) or Central-West (15.9%) regions of the country. Hotels and hostels (rented or provided by an employer) were the most commonly reported types of accommodations (34.9%). For 1217 (46.0%) of the consultations, travellers estimated that the length of their stay would be ≤30 days (Table 2). Of the 2836 consultations, 901 (31.8%) were conducted within 7–21 days before the travel.

**Table 2:** Characteristics of the travel population assisted at the Instituto Emílio Ribas travel clinics, São Paulo, Brazil, between January 2006 and December 2010

Variable (completeness)	N	%
*Travel reason (99.0%)*		
Study or work	1480	52.7
Tourism	1013	36.1
Multiple or others	316	11.2
*Travel destination (98.8%)*		
Africa	694	24.7
Asia	417	14.9
Brazil	422	15.1
Others	1270	45.3
*Accommodation type (86.2%)*		
Hotel or hostel	854	34.9
Others	1590	65.1
*Length of stay (in days) (93.3%)*		
≤30	1217	46.0
31–180	805	30.4
>180	624	23.6

### Malaria prevention recommendations

Prophylaxis against malaria was recommended in 292 of the 2836 consultations (10.3%). The most prescribed chemoprophylaxis regimens were doxycyline (210/71.9%), atovaquone-proguanil (49/16.8%), mefloquine (27/9.2%) and chloroquine (6/2.1%). Stand-by emergency treatments for malaria were recommended for 34 of the orientations (1.2%). All travellers were advised on personal measures against mosquito bites.

### Factors associated to malaria chemoprophylaxis recommendation

Table 3 summarizes the results of the univariable analysis evaluating factors associated with chemoprophylaxis recommendation. Male gender [OR 1.37 (95% CI 1.07–1.75)]; age < median [OR 1.34 (95% CI 1.05–1.71)]; African destinations vs Asian [OR 4.62 (95% CI 3.07–6.95)]; Brazilian [OR 4.67 (95% CI 3.11–7.04)]; and other destinations [OR 9.12 (95% CI 6.53–12.74); and length of travel stay ≤30 days [OR 3.33 (95% CI 2.17–5.11)]; were associated with the higher odds of chemoprophylaxis recommendation. Schooling ≥12 years (vs <8 years) [OR 0.47 (95% CI 0.23–0.98)]; and tourism travels (vs work travels) [OR 0.72 (95% CI 0.56–0.94)]; were associated with the lower odds of chemoprophylaxis recommendation.

**Table 3:** Univariable analysis to identify factors associated to malaria chemoprophylaxis recommendation among individuals attended at the Instituto de Infectologia Emílio Ribas, in São Paulo, Brazil, from January 2006 to December 2010

Variables	Chemoprophylaxis recommendation		OR (CI 95%)
	Yes (n; %)	No (n; %)	
*Gender*			
Male	163 (55.8)	1220 (48.0)	1
Female	129 (44.2)	1324 (52.0)	1.37 (1.07–1.75)
*Age group*			
<Median	165 (56.5)	1253 (49.3)	1
≥Median	127 (43.5)	1291 (50.7)	1.34 (1.05–1.71)
*Schooling (years; 90.3%)*			
<8	8 (2.9)	128 (5.6)	1
8–11	40 (14.7)	447 (19.6)	0.70 (0.32–1.53)
≥12	225 (82.4)	1706 (74.8)	0.47 (0.23–0.98)
*Any immunosupressive condition*			
No	286 (97.9)	2483 (97.6)	1
Yes	6 (2.1)	61 (2.4)	1.17 (0.50–2.73)
*HIV infection*			
No	290 (99.3)	2512 (98.7)	1
Yes	2 (0.7)	32 (1.3)	1.85 (0.44–7.75)
*Diabetes mellitus*			
No	288 (98.6)	2510 (98.7)	1
Yes	4 (1.4)	34 (1.3)	0.97 (0.34–2.77)
*Depression*			
No	283 (96.9)	2459 (96.7)	1
Yes	9 (3.1)	85 (3.3)	1.87 (0.54–2.18)
*Travel reason*			
Study or work	137 (47.2)	1343 (53.3)	1
Tourism	125 (43.1)	888 (35.3)	0.72 (0.56–0.94)
Multiples or others	28 (9.7)	288 (11.4)	1.05 (0.68–1.61)
*Travel destination*			
Africa	183 (62.9)	511 (20.3)	1
Asia	30 (10.3)	387 (15.5)	4.62 (3.07–6.95)
Brazil	30 (10.3)	392 (15.6)	4.67 (3.11–7.04)
Latin American endemic countries	11 (3.8)	205 (8.2)	6.67 (3.55–12.53)
Others	37 (12.7)	1017 (40.5)	9.84 (6.81–14.23)
*Accomodation type 2512*			
Hotel or hostel	79 (32.8)	775 (35.2)	1
Others	162 (67.2)	1428 (64.8)	0.90 (0.68–1.19)
*Length of stay (in days)*			
≤30	154 (54.2)	1063 (45.0)	1
31–180	104 (36.6)	701 (29.7)	0.98 (0.75–1.27)
>180	26 (9.2)	598 (25.3)	3.33 (2.17–5.11)

*CI* confidence interval

African destinations vs Asian [AOR 5.01 (95% CI 3.23–7.14); Brazilian [AOR 4.76 (95% CI 3.13–7.14)]; and other destinations [AOR 8.33 (95% CI 5.88–12.50)]; and length of travel stay ≤30 days [AOR 2.63 (95% CI 1.67–4.00)]; were independently associated with the higher odds of chemoprophylaxis recommendation after the logistic regression (Table 4).

**Table 4:** Variables independently associated with the recommendation of malaria chemoprophylaxis in pre-travel consultations among individuals attended at the Instituto de Infectologia Emílio Ribas, in São Paulo, Brazil, from January 2006 to December 2010

Variable	AOR	95% CI
*Destination*		
Africa	1	–
Asia	5.01	3.23–7.14
Brazil	4.76	3.13–7.14
Latin American endemic countries	6.14	4.23–8.50
Other	8.33	5.88–12.50
*Length of stay (days)*		
≤30	1	–
>180	2.63	1.67–4.00

*AOR* adjusted odds ratio, *CI* confidence interval

## Discussion

Currently, available malaria transmission indicators are important tools for the assessment and understanding of the risks of disease transmission in populations of endemic regions. However, these values among individuals moving from non-endemic areas for malaria transmission areas are little known [15]. Non-immune migrants, mobile populations and travellers population groups are at considerably higher risk of contracting malaria, and developing severe disease, than populations living in endemic areas [1, 3]. Furthermore, stronger malaria surveillance and prevention systems are urgently needed to enable a timely and effective malaria response in eliminating areas for preventing reintroduction. The policy of prevention of malaria recommended by the World Health Organization is to recognize differencial risks of transmission, personal measures avoiding mosquito bites, early diagnosis and treatment, chemoprophylaxis and/or self-administered treatment when indicated [3].

In this work, most of the travellers were adults with high educational level travelling by work or study reasons. When travellers were asked how they had become aware of the travel medicine service, the most common responses were from suggestions of friends, health professionals and electronic media. Agricultural and mining projects attract many temporary workers to the Brazilian Amazon, which may return to their places of origin carrying malaria [18]. Destinations of the study population to Africa and locations within North or Central-West Brazilian regions were common.

Most of the cases diagnosed and reported outside the Brazilian Amazon corresponded to imported cases originating from the Amazon or other Central and South American, African or Asian countries that have active transmission [19]. Interestingly, previous studies performed in areas where malaria transmission does not occur, showed *P. vivax* relapses rates ranging from 25% over 7 years in São Paulo [20] to 40% over 6 years in Rio de Janeiro [21] in individuals returning from endemic areas. A delay in diagnosis and treatment, common in areas where the disease is not endemic, can result in serious illness and death, also proportionately more common in non-Amazon region [22]. Probably, the bigger number of people with African destinations searching for medical travel services is influenced by the massive report by the Brazilian press of deaths in patients with falciparum malaria returning from Africa to non-endemic regions of the country [23, 24].

Prophylaxis against malaria was recommended in 10.3% of the consultations. Consistently, in another Brazilian study, authors reported that such prophylaxis was recommended for 9.1% of the travellers evaluated in their study [25], suggesting that vast majority of the travellers do not present a considerable risk of acquiring malaria in their destinations according the medical evaluation. The decision to use chemoprophylaxis on travellers depends on the individual’s risk–benefit analysis, weighing the risk of contracting malaria against the possible adverse effects of anti-malarial medications, underlying health conditions, duration of travel, adherence, cost and finally the licensed status of the anti-malarial medications [15, 26, 27]. Brazilian official guidelines also include the risk of *P. falciparum* malaria as a major issue in the decision of recommending the use of chemoprophylaxis [15].

In this study sample, the prophylactic drug most often prescribed was doxycycline (71.9%), followed by atovaquone-proguanil (16.8%). In the study conducted by Wilder-Smith et al., doxycycline was also found to be the most widely prescribed anti-malarial drug [28]. According the Brazilian guideline, there is no consensus about the best chemoprophylactic regimen to be used in each particular case [15]. Doxycycline was suggested as a good chemoprophylaxis for short-term travellers at particular risk of chloroquine/proguanil resistant *P. falciparum* malaria [29]. However, chemoprophylaxis failures have been related to a lack of compliance with doxycycline due to its short elimination half-life [30]. Atovaquone-proguanil is at least as efficient as mefloquine [9]. Mefloquine was suggested as the elective chemoprophylaxis for long-term travellers at particular risk of chloroquine-resistant *P. falciparum* malaria [31]. Regarding safety, a systematic review of randomized and quasi-randomized controlled trials showed that atovaquone-proguanil and doxycycline are the best tolerated regimens, and mefloquine is associated with adverse neuropsychiatric outcomes [32]. As mefloquine and atovaquone-proguanil are not available for purchase nor is part of National Malaria Control Programme in Brazilian territory, their use by travellers was highly limited.

Travel to African destinations was the most important factor associated to malaria chemoprophylaxis recommendation. Again, this finding may be explained by the recommendation from Brazilian guidelines to use chemoprophylaxis by travellers visiting *P. falciparum* endemic areas [15]. On the other hand, the large contribution of *P. vivax* infections in Asia and Latin America, including the Brazilian Amazon [33], explains the low chemoprophylaxis prescription to travellers visiting these areas. Today, *P. vivax* accounts for 84% of registered cases of malaria in Brazil [2]. Previous studies had already demonstrated a tendency in the reduction of the recommendation of chemoprophylaxis for travellers with destinations to the Indian Subcontinent and Latin America where *P. vivax* predominates [14, 34]. However, *P. vivax* causes relapses [35, 36] and potential severity [35, 36], changing the old paradigm of not considering prophylaxis in those areas [16]. Regarding the chemoprophylaxis for vivax malaria, the Brazilian policy is not to make this recommendation [15], due to all the complexities involved for its practice. Chemoprophylaxis for *P. vivax* requires essential measures such as G6PD status assessment, not always practical in travel medicine services.

It was expected in this work that pre-travel preparation of travellers with immune suppression due to any medical condition would be a greater concern regarding malaria chemoprophylaxis. Immunocompromised travellers to malaria-endemic areas should be prescribed malaria chemoprophylaxis and receive counseling about mosquito bite avoidance [37]. Special concerns for immunocompromised travellers include any of the following possibilities [37]: (a) drugs used for malaria chemoprophylaxis may interact with drugs in the traveller’s maintenance regimen; (b) the underlying medical condition or immunosuppressive regimen may predispose the immunocompromised traveller to more serious disease from malaria infection; (c) malaria infection and the drugs used to treat malaria infection may exacerbate the underlying disease; and (d) the severity of malaria is increased in HIV-infected people.

Recommendations for malaria chemoprophylaxis involve complex decision-making and must consider the destination, the host, the activities, and the duration of exposure. The choice of medication depends on the risk of malaria at the destination, resistance, the profile of the traveller (contra-indications, underlying health conditions, purpose of travel such as travellers visiting friends and relatives (VFR), the duration of travel and finally cost and adherence issues. The registration status of the anti-malarial medications is another factor.

Malaria treatment policy in Brazil is guaranteed by the government. Therefore, anti-malarial drugs are distributed free of charge only by reference public services in the country. An ideal anti-malarial for malaria prevention is the one of lower cost, with less risk of causing adverse events, with easy access and easy dosage schedule.

The association atovaquone and proguanil could be the ideal choice for travellers but because of its high cost, its inclusion in the therapeutic arsenal of Brazil is questionable.

A coherent measure of malaria prevention in Brazil and for international travellers would be to recommend for all parts of Northern Brazil, avoidance of mosquito bites and immediate consultation of a physician in case of fever during or after the journey is recommended. Obviously, the accurate evaluation of the risk of the traveller in acquiring malaria in the Brazilian Amazon, chemoprophylaxis may be indicated.
