# Erratum to: Pre-travel malaria chemoprophylaxis counselling in a public travel medicine clinic in São Paulo, Brazil

**DOI:** 10.1186/s12936-017-1739-6

**Published:** 2017-03-17

**Authors:** Tânia do Socorro Souza Chaves, Wuelton Marcelo Monteiro, Jessé Reis Alves, Marcus Lacerda, Marta Heloisa Lopes

**Affiliations:** 1 Parasitology Section, Evandro Chagas Institute, Rodovia BR-316, Km 7 s/n, Levilândia, Ananindeua, PA 67030-300 Brazil; 2 Travel Medicine Division, Emilio Ribas Institute of Infectious Diseases, Av. Doutor Arnaldo 165, Cerqueira César, São Paulo, 01246-900 Brazil; 3 Departamento de Doenças Infecciosas, Faculdade de Medicina da Universidade de São Paulo, Av. Dr. Arnaldo 455, Cerqueira César, São Paulo, SP 01246-903 Brazil; 4 Fundação de Medicina Tropical Dr. Heitor Vieira Dourado, Manaus, Brazil; 5 Universidade do Estado do Amazonas, Manaus, Brazil; 6 Instituto Leônidas & Maria Deane (Fiocruz-Amazônia), Manaus, Brazil; 7 Disciplina de Doenças Infecciosas e Parasitárias, Faculdade de Medicina, Universidade Federal do Pará, Av. Generalíssimo Deodoro, 01-Umarizal, Belém, PA 66050-160 Brazil

## Erratum to: Malar J (2017) 16:64 DOI 10.1186/s12936-017-1713-3

After publication of the original article [1], it came to the authors’ attention that Fig. 1 was annotated in Portuguese. The version of the Figure created using English is published in this erratum (Fig. 1).

**Fig. 1:**
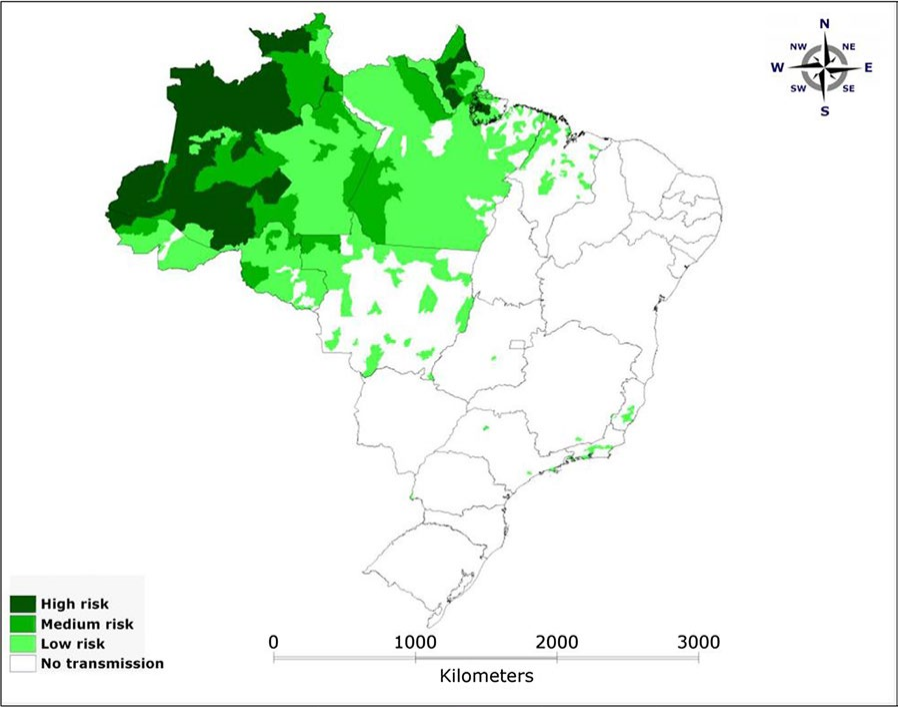
Areas at risk of malaria transmission in Brazil [2]. Source: National Malaria Control Programme/Ministry of Health of Brazil
